# Promoting Pluralism in Counselling: an Untapped Source of Relational Mapping as Therapeutic Process

**DOI:** 10.1007/s10447-017-9298-7

**Published:** 2017-07-21

**Authors:** Donna Carlyle

**Affiliations:** Faculty of Health and Life Sciences (Social Work, Education and Community Well-being), Northumbria University, Coach Lane Campus, Newcastle-upon-Tyne, NE7 7AX UK

**Keywords:** Hybridity, Pluralism, Child/human development, Psychogeography, Mapping, Diagramming

## Abstract

This paper discusses the merits of pluralism in practice. It argues for a wider recognition of creative and integrative approaches, such as those used in the field of children’s geographies (involving places and spaces), as a way of unlocking practitioner potential and innovation. By re-thinking child and human development, viewing it as socially, culturally and philosophically bound, through the proposed concept of *‘vectors of entanglements’*, the author seeks to demonstrate and encourage the application of hybrid approaches across multi-disciplinary fields. Through the use of diagramming and mapping the interconnectedness of relationships across space and place, the therapeutic process is brought to life to encourage practitioners to explore the ‘invisible’ threads that constitute significant meanings to clients.

## Introduction

Considerations of attachment theory and Bowlby’s (1979) significant contribution to the field of child development have shaped our understanding of children’s interactions with others, as being biologically and evolutionarily predetermined. This has been further endorsed by neuroscience through advances in mapping and imagery of the incredible exponential wiring of the human brain (Balbernie 2001). Such advances can now reveal the ‘bundle of lines’, entwined and overlapping, that constitute the sculpture of the brain. Such visual evidence has proven unequivocal in terms of the relational aspects in regard to brain wiring and the billions of connections that need to be made by the young infant in early life. Hence, such awareness is being embraced into policy, practice and early intervention and support for children across disciplines (Allen 2011).

The notion that we are all ‘bundles of lines’ is a very creative and contemporary idea, and a new synthesis of thought is required to bring forth such illuminating concepts to ensure greater theoretical integration into the understanding of human development and to inform practice. Such integration affords a less sequential trajectory of child development previously assumed, and re-positions a child’s individuation and creativity by considering them as *‘vectors of entanglements’;* as corporeal, visceral and vitalistic bodies that create relations of production through complex chains of interactions, interconnections and entanglements through webs of relationality:

…we are all made of lines. We are not referring to lines of writing. Lines of writing conjugate with other lines, life lines, lines of luck or misfortune, lines productive of the variation of the line of writing itself, lines that are *between the lines* of writing.

(Deleuze and Guattari 1987, p. 228

As Bronfenbrenner (1979) asserted, the environmental world matters. Children gain their sense of emotional security largely from their relationship with their caregiver/s; but this in itself, is configured in an existing and ongoing dependence of the caregiver/s on the natural world. If we were to *see* these multiple lines and transactions (transactional processes with reciprocal effects across time between child-parent/s), we would observe a continuous flow of interactions among the child-parent/s-environment dynamics (Sameroff and Chandler 1975).

## The Promise of ‘Being’ and ‘Becoming’ Hybrid

Current reliance on cognitive explanations to sensing and feeling has somewhat marginalised and undermined human well-being and potential, and Kidner (2007) has cautioned that this narrowed focus would be ecologically catastrophic. It reinforces the divide and dualism of nature/culture, thus limiting wider perspectives that enable creativity and autonomy.

Existentially, this could also involve an annihilation of vast ontological proportions in its discounting and disregarding of multiple subjectivities and multiple ways of being in the world. This is particularly pertinent for children who can relate and communicate powerfully without linguistic prowess, and who ooze an energy and vitality that is affective and pre-cognitive. Therefore, a pluralistic and hybrid approach, as developed in children’s geographies (Whatmore 2002), could steer child counselling in a new and important direction, enriching current practice in innovative and exciting ways. Such an approach would enable the appreciation of corporeal, embodied and aesthetic-visual aspects of experience to be more fully incorporated into counselling practice.

Similar directions have become evident within the fields of counselling and psychotherapy. Cooper and McLeod (2007), in attempting a synthesis of multiple theoretical approaches, referred to by them as a ‘pluralistic meta-theory’, have stated (p. 5):

Unlike singular models and systematic forms of integrationism, a pluralistic framework is open to an infinitely wide range of ways of engaging with individual clients. Unlike an eclectic approach, however, the pluralistic meta-theory provides a framework through which this multitude of practices and conceptualisations can be organised, contrasted and evaluated.

However, practitioners have yet to fully grasp the opportunity of such a tantalising journey into a new place of pluralism and hybridity. By considering the example of psychotherapy, Dryden’s (2012) critical argument for pluralism to be ‘married’ to the ‘working alliance’ carries considerable challenge, as well as opportunity. But there are far greater concerns that may be stifling progress to this new land.

The continued endorsement of unitary approaches, such as cognitive-behaviour therapy, systemic, psychodynamic and humanistic therapies, by such bodies as the UK Department of Health (2001) and its IAPT (Improving Access to Psychological Therapies), has not aided this transition smoothly. Practice remains in roles that are theory-bound ‘silos’ creating inevitable tensions and divisions. This is despite the fact that research and ‘evidence-based’ knowledge concludes that most modalities can be regarded as being largely of equal merit (Wampold 2001; Wampold and Imel 2015). There are excellent examples of how this integration can work, such as that of Cognitive Analytical Therapy (CAT), which combines cognitive and psychodynamic approaches (Ryle et al. 1990), and systemic and cognitive-behavioural modes (Dummett 2006). A post-structuralism philosophy could transcend the boundaries of theoretical and professional resistance, opening up the field to research and practice in pluralistic approaches.

By using suggested frameworks such as that purported by Cooper and McLeod (2007) and their idea of process maps (mapping and reviewing the individual’s therapeutic process and journey), there is vast potential to link the relational and messy endeavour of collaborative practice to a visual understanding and a visual vocabulary. This can support individuals to use a means of expression other than language. It is such lines and process maps that afford the means for a dynamic interaction of self-discovery and attunement. As Ingold (2000, p. 20) pointed out, “in a relational model kinship is geography.” This suggests that the environment around us, as well as the interpersonal relationships we experience (such as with our main attachment figure/s, for example) are inextricably linked and inseparable. This relational field is the ground from which things grow and become.

There is no discontinuity between the self and the ground, but, rather, the connection can be regarded as an extension of self. Deleuze and Guattari (1987) notion of the rhizome (an underground stem) being rooted firmly in order to grow and flourish offers a metaphor in this regard. Rhizomes can be akin to synaptic neural pathways that, in similar vein, stretch and connect, fired by an ‘actualising’ tendency (Rogers 2002) or ‘lines of flight’ as postulated by Deleuze and Guattari (1987). It is here that Deleuze and Guattari (1987) could be considered to challenge neuroscience, in that ‘hard wiring’ of the brain suggests a fixed identity or attachment pattern (although brain plasticity is acknowledged), in contrast to their idea of continued ‘being’ and ‘becoming’, which, like Rogers’ concept of ‘becoming a person’, is a fluid and ever changing process. Schon (1984), in his seminal work on reflection in practice, further considered that, only by entering the ‘swampy, messy lowlands of practice’ can we learn to reflect-in-action. Patterns then emerge to inform *intra-action*, enabling an inner closeness (which is subtly different to attachment) fundamentally becoming attuned together and being affected by one another, creating multiple subjectivities (Barad 2007; Stern 1985).

Perhaps by entering a more hybrid paradigm of practice, one in which a visual vocabulary and affective encounters can be allowed to take place (and space) along creative lines, we can incorporate new, challenging and exciting ideas into our therapeutic engagements. These can take on the shape of rhizomatic maps, movements and diagrams; thus, tracing a journey that travels along a mutual path of discovery. Pure modalities prevail, yet, because evidence informs us that not ‘one size fits all’, a pluralistic and integrative perspective is advocated. We continue along our familiar unitary paths for positive ‘outcomes’ to our peril, burdened by the weight of evidence-based practice demands. What we lose along the way is the creative ‘self’ we possess.

The tendency to categorise, hypothesise, label and construct definitive ideas of human behaviour is largely a manifestation of our benevolent quest to heal and help. This type of classification is exposed by both Deleuze and Guattari (1987) and by Latour (1993) as being unhelpful, reductive and regulating. If we re-think the making of connections to others and the world around us, we can begin to exercise a relational approach that eradicates such a tendency. There are amazing visual and dynamic visceral forces at play, which we emit in our interactions with one another. These affective capacities enmesh and entwine with one another, meaning that we are able to affect and be affected by others, both human and non-human (such as involving objects, artefacts, architecture, animals, and such like). Bennett’s (2010, p. 20) notion of ‘thing power’ illuminates this very well in her delightful description of vibrant matter and ‘materiality’s’ visceral nature.

The power of objects and things to convey a force and vitalism has resonated with several other scholars. We only need remind ourselves of how Freud gained tremendous insight from watching his grandson (Ernst) playing with a simple bobbin reel, observing how he would throw the bobbin behind a curtain and say ‘Ooooh’ and then, retrieving it by pulling on the string, he would say ‘Aaaah’. Freud deduced that his grandson was ‘testing out’ what it means to go away and come back – separation and reunion. This symbolic re-enactment of the coming and goings of Ernst’s mother swayed Freud to go ‘beyond the pleasure principle’ (1920) in his realisation that loss and recovery constitute both pain and pleasure (Freud 2010; Lapsley 2013). What Ernst could also be considered to be demonstrating were his lines of flight – his bolts of creative energy between separation and individuation (Duchinsky et al. 2015). This is also in keeping with Deleuze and Guattari’s (1987) view of the child being a vector of the process of returning-to-oneself-as-an-other. The exploration of venturing forth (throwing the bobbin reel) and returning it safely back to himself could be seen as representing Ernst’s playful experimentation with growth.

Along similar lines, Winnicott (2008), in his use of the ‘Squiggle’ drawing (a simple ‘squiggly’ line drawn on paper that can serve as a catalyst for a child’s creative expression), showed how he entered the emotional minds of children to open up new ways of communicating with and supporting them. He expanded this notion of lines and scribble, entering the child’s world - a realm rich in imagination and wonder - eliciting the power of the unspoken and invisible. His classical account of his work with the young girl nicknamed ‘the Piggle’ (Winnicott 2008) represents the boundless creativity he tapped into in order to understand and attune to children’s emotional and relational landscapes. Both Freud and Winnicott’s conceptions became the cornerstone for some of their most influential work; each in their own account highlighting the wonderfully creative ways we can understand and support growth and development. They demonstrate the ‘lines of flight’ of being and becoming, towards observing the child’s engagement in the world with awe and enchantment.

## The Multiplicity of Children

What both Freud and Winnicott’s examples have in common could be revisited through Ingold’s (2016) captivating idea of ‘lines.’ In taking the seemingly innocuous concept of how we create lines though movement, Ingold provides juxtaposition between Deleuze and Bowlby, and Deleuze and psychoanalysis. Through this anthropology of lines we can begin to explore more of the multi-faceted nature of being; the sensory and embodied experience that is often given less credence within a dominant linguistic and cognitive discourse. If we re-think children towards being vectors of affect, *vectors of entanglements*, then we are invited into a world of ‘meshwork’ in which relationships can be woven into rich tapestries of new meaning and relational fabric. The reciprocal ‘flows’ between children and others (including objects, things, animals and ‘stuff’) takes place through sensory awareness and along lines of connectivity. Indeed, the symbolic image of the child’s teddy bear encapsulates this phenomenon wonderfully in how it comforts and soothes, materialistically and emotionally. The corporeal ‘dance’ of teddy (not merely as a transitional object) and child takes place through movement, linear entanglements, without beginning or end, along ‘lines of flight’ that can be considered multiple ‘trails of becoming’.

This tactile encounter as metaphor is transferrable to the counsellor and child relationship, allowing them to be ‘in touch’ with and connect to both the internal and the external worlds of each other within a transitional space (Bingley 2003). Ingold (2006, p. 53) captures this beautifully and asserts, “life will not be contained but rather threads its way through the world along the myriad lines of its relations”. In this sense, we can dislocate childhood from psychological conceptual models and return to ‘a means of knowing’ that interrogates the dominant discourse of attachment theory; not replacing but *enhancing* it. Patterns of interactions *are* patterns of lines entwined and woven together. A sensory process emerges in banal encounters, reframing them as meaningful and vital. A new kind of ‘object-relations’ can emerge in counselling practice; one in which there is greater integration and recognition of ecological ontology and human geography. These patterns, tracings and trails can be considered the remnants of ourselves that we infuse and leave behind; our affect and energy poured into spaces and atmospheres (Griffero 2010). Even Freud revised his concept of ‘primary narcissism’ once he understood that the infant was not just a ‘bundle of lines’ driven by innate drives and urges, but also by a need to *connect*. As such, Freud was something of a pluralist, he did not lack the courage to change his mind and consider other possibilities.

Rather than just plotting a child’s developmental milestones and growth on centile charts, or considering their attachment pattern, we can increase our awareness of their inner states by mapping, or diagramming, how they grow and connect in new ways (through observing encounters with ‘others’ or ‘agents’ around them). The different individuated relations a child enters can enable greater understanding of how a child ‘becomes’ and grows. These ‘agents’ are not always persons (as indicated, they can be toys, teddy bears, pet animals, for example) and they function and serve as a basis of relations – relations of production. Process mapping, tracing or diagramming can serve as a tool for pluralism to emerge in therapeutic encounters. These encounters can be sensory produced and not exist merely in an abstract domain.

New approaches to research are opening up a new way of exploring identity formation and how the child can be *rhizomatic;* that is, truly being and becoming*.* This can be transferrable to the practice of counselling. Beyond Deluzian metaphor and neologisms, the kinaesthetic and visceral logics of the child’s body has significant meaning. As Freud postulated the fundamental aspects of the human psyche as involving primitive drives and desires, we should awaken new configurations by synthesis across scholarly ideas. By adding Deleuze and Guattari's (1987) concepts of human development into the mix, we can consider a corporeal model of experiencing life and subjectivity outside of stratification. Ingold’s lines complement and accentuate this image, creating visual and virtual maps of how we *dwell* in the world and engage in our environment. This ontology of dwelling offers not only a culturally sensitive lens through which to view the world, but also a unified approach among psychology, anthropology, and ecology.

In keeping with this, we can consider Ingold’s use of the term *interagentivity* rather than intersubjectivity (Ingold, 1992, p. 47). This enables us to appreciate the flow and flux of our interactions, so succinctly described by Deleuze and Guattari (1987, pp. 237–238):

Individual or group we are traversed by lines, meridians, geodesics, tropics, and zones marching to different beats and differing in nature…The lines are constantly crossing, intersecting for a moment, following one another…It is an affair of cartography. They compose us, as they compose our map. They transform themselves and may even cross over into one another. Rhizome.

## Mapping the Route to Pluralism

**Fig. 1:**
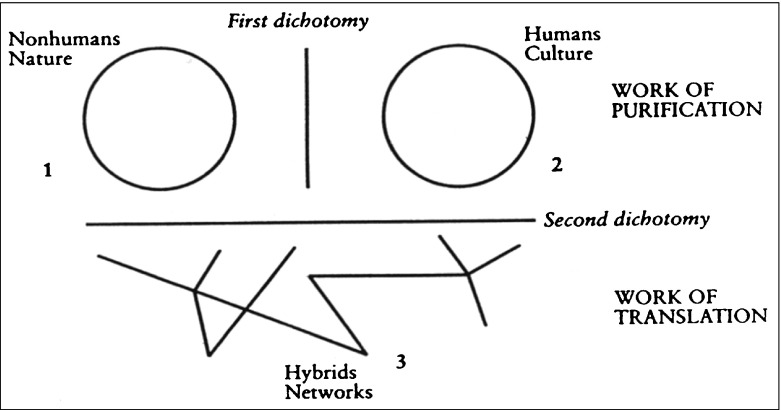
Purification and translation elements (taken from Latour 1993)

As Bondi (1999) suggests, we need to reconcile psychotherapeutic methodologies and geography, thus allowing for broader methodologies with which we conduct research, in addition to widening pluralisms in practice, which can also encompass hybridity. It is through the synergy of broader methodologies that pluralisms in practice will flourish and emerge. Whatmore (2002) invites the adoption of ‘relational human geography’ by exploring the ebb and flow of the energetic world. Her idea of ‘hybrid geographies’ is both inspiring and transferrable across disciplines. She provides a context for multiple ways of knowing in using Latour’s (1993) ‘purification and translation’ diagram (see Fig. 1).

Whatmore (2002) draws attention to how we need to take note of the hybrid networks ‘under the line’ of rational and conceptual boundaries. This helps to decipher, counter-act and reject the complex ambiguity of Cartesian duality; mind/body, subject/object, nature/culture. Such dichotomies further endorse ‘pure’ modalities in practice and stifle the potential of pluralism to emerge, infuse, permeate and transform creative thinking. As Latour’s (1993) diagram implies, an allegiance with Deleuze’s idea of the rhizome seems evident. Through purification, classifications are produced and dichotomies created, thus nature and nurture become separated. In the hybrid network of translation, he extrapolates how all things are connected (as indeed is all knowledge), and as such they are not separate or unitary.

**Fig. 2:**
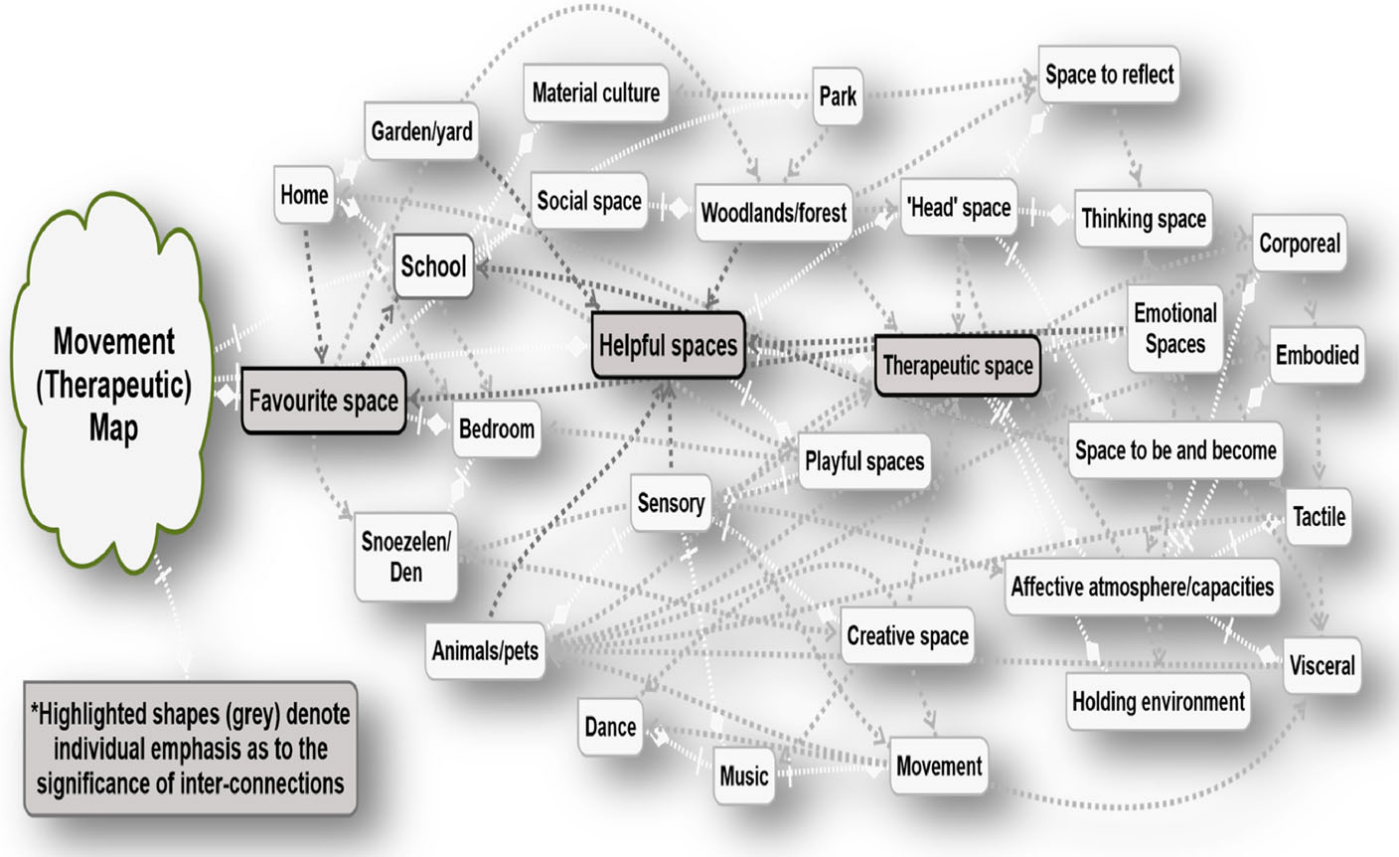
Example Rhizomapping to show lines, connections and entanglments

**Fig. 3:**
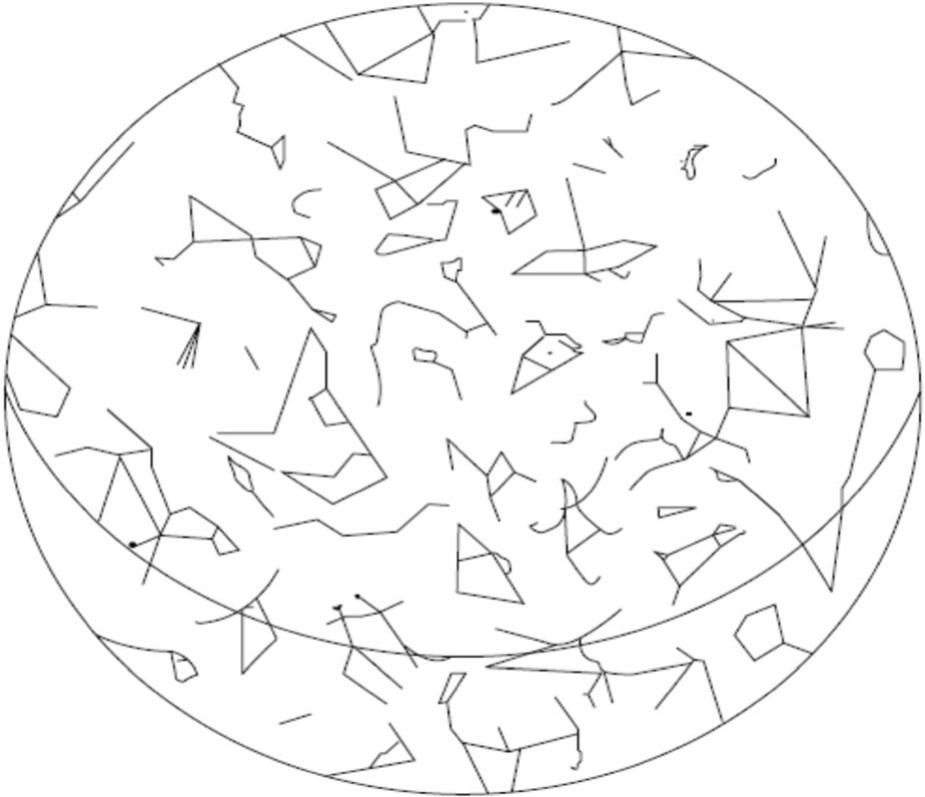
Constellations of the northern celestial hemisphere (taken from Ingold 2016, p. 51)

By capturing everyday activities and entanglements through synchronising with maps, drawings and lines, the emotional landscape can be seen in all its veracity. Therapeutic elements involved in the production of Movement Maps (see Fig. 2 as an example) can be a visual capturing of this and many other aspects of relational landscapes. The lines involved can be viewed as ‘ghostly lines’ – human-made lines of movement lacking material substance or form but existing in our conceptual worlds (Ingold 2016, p. 50). As he evocatively suggests, “looking up at the night sky we imagine the stars to be visibly connected by ghostly lines into constellations” (Ingold 2016, p. 50; see Fig. 3).

As Whatmore (2002, p. 6) highlights, in regard to theoretical claims or universal designs, “hybrid mappings are necessarily topological, emphasising the multiplicity of space-times generated in/by the movements and rhythms of heterogeneous association”. This invites counsellors and psychotherapists to unsettle the current co-ordinates that direct practice, encouraging a common ground, and a new terrain of pluralism.

This is further endorsed by Rose (2016, p. 23) who asserts that “any account of being human that reduces an individual to a singleton in a world of singletons is missing the point. We are who we are in the context of others”. Hence, if we marry Ingold’s anthropology of lines to psychogeography and counselling we can begin to map a route towards pluralism in counselling practice. By considering our walking, movement, habitus and the traces and trails of these actions, we can begin to foreground and merge differing paradigms in new and lively ways.

This prospect is not merely an abstract or esoteric concept, but is applicable and transformative to the moment-to-moment therapeutic encounter. As such, the importance of affect is key in understanding how feelings are poured into therapeutic spaces where the presence of empathy, for example, is received and felt between bodies (counsellor and client). These are feelings (entangled) transmitted through the body, as a sensory and visceral force of energy flow, involving invisible threads that connect and bind us (Simmel 1997). It involves an emotional atmosphere charged with individual engagement, experience and endeavour, which constitutes Winnicott’s (1971, p. 107) ‘potential space’ (analytic space, cultural experience or area of creativity). A therapeutic relational depth is created in the space, which is transformed into invisible threads, to convey the rhizomatic weavings of the counsellor alongside the client. These threads (or lines) can be made visible (materialisation of affect through drawings) and thereby enliven the relational encounter in a dynamic and creative way.

Within such entanglements, empathic communication shifts the client into a new and different space as they integrate this meaningful experience. As such, the mapping of process is animated and brought into liveliness through its visual capture. This in itself can be validating and transformative for the client as experience/encounter is both felt and seen. Therefore, mapping and diagramming the therapeutic process provides a powerful tool, a reflexive measure of emplacement (understanding of the relationship between body and the environment) (Pink 2011). This further endorses the ideas of Ingold (2000) in that human life is fundamentally based on or in (physical) movement and is bound up with our environment and how we come to ‘know’ourselves. He describes this in detail (Ingold 2000, p 229):

Knowing, like the perception of the environment in general, proceeds along paths of observation. One can know *in* places [rather] than travel in them. Rather, knowledge is regional; it is to be cultivated by moving along paths that lead around, towards or away from place, from places elsewhere.

By thinking of places being experienced as embodied, as atmospheric, infused with activity of differing intensities (emotions and affects) and presence, place can become an ‘event’ (Pink 2011). Such events are worthy of being traced, mapped and seen in order to assist children/adults in bringing into awareness (feeling or articulating) complex emotions that can sometimes be difficult to be in touch with.

## Discussion

What both Winnicott and Freud’s earlier examples demonstrate are the visceral connections and sensorial nature of being, which are purported to be stronger in childhood (Sibley 1995). As Causey (2017) asserts, the drawing (of lines) can connect us to our ‘inside’ and ‘outside’ worlds through the capturing of embodied memories and experiences. These ‘visual stews’ help us to re-create shapes, forms and patterns that make up our sense of place and space, without translating the moments to words (Causey 2017, p. 8). By using this method, counselling practice can be enriched with visual curiosity. As Robson (2016) purports in his practical application of aesthetic-visual methods with students, this curiosity can enable further learning and reflection. Pluralism requires more than integration of methods and approaches. It requires an inter-disciplinary approach to nudge counselling along the path of change and transformation. By engaging in this creative line making, we begin to see shared connections and perspectives, both between client and counsellor as well as between theory and philosophy.

It is within the context of therapeutic space that children negotiate the tensions between regulation (of space) and individuality. As James (1993, p. 27) pointed out, children’s perceptions of their own body and spatiality are a significant source of ‘identity and personhood’. Therefore, by foregrounding children’s embodied and visceral connections a new hybridity can emerge and unfold – thereby revealing graphically and visually the promise of pluralism. As child health and development encompass cross-disciplinary fields, the accommodation of pluralism to practice can offer a rich source of understanding the complexity of relational processes.

The production of lines can depict many diverse feelings and, as both Ingold (2000) and Causey (2017) highlight, we can materialise affects through adopting this visual vocabulary. This embodied encounter can enliven our work and offer a richness to self- expression, aiding its articulation into material form and being. This in itself is an integrative and transformative process for the individual, and one that can be ongoing and helpful.

## Conclusion

Lines are everywhere. They are already embedded into everyday practice, depicted in genograms, ethograms, timelines, and ecomaps, to name but a few. Perhaps Deleuze, Guattari, and Ingold have provided a ‘life-line’ to research and practice. It can take many shapes and forms, and it is only the limitations of our imagination that renders them insignificant, intangible and invisible.

By reconciling and synthesising a myriad of approaches, new lines of inquiry can emerge. As children direct their attention as individual agents who respond to objects as affordances within their environments (see Gibson 1979), so too can professionals by becoming as equally playful and creative with knowledge. In exploring new ideas, we learn a new language. This new language draws upon the ideas of attachment theory and commonalities between ethology and new materialism. By attempting such an immense exposition and reflection on the movement of human individuation, discourses that place children in negative relationships with others deemed unreliable and untrustworthy can be debated and critiqued. Dominant discourse can become multiple discourses. Theories and ideas can become integrated in their differences and similarities. Prevailing paradigms such as those of sociological study being underpinned by cognitive, verbal facility and language (Edwards 1997) can be considered in light of the complexity of social interactions between human and non-human objects/subjects.

Through embracing this diverse, opportune, pluralistic, hybrid and philosophical position, we become open as human beings, to be counsel to all the vicissitudes of our own ‘knowing’ and ‘becoming’. Such lines of flight can be enchanting and liberating to practice. This offers therapeutic approaches a shared, visual landscape; a space and place for new connections and entanglements to emerge and unfold. In doing so, pluralism can find a platform that allows individual growth, self-reflection and self-awareness as a joint parallel process between counsellor and counselled. This reaches beyond current practice restrictions and returns it to being needs-led rather than outcome-led. Pluralism matters.

We cannot ‘unknow’ what we already know, or be entirely ‘pure’ in our practice. Can counsellors merely focus just on an individual’s ‘unhelpful cognitions’ without knowing their systemic as well as schematic nature? All theories are inter-linked and entangled, not separate and disparate entities. We must take up the challenge of ‘hybridity’ and pluralism through mingling and merging with all our capacities as ‘psycho-geographers’ (Rose 2016, p. 23) to share and shape what it means to be alive and dwell in the world. There are many paths to walk, many maps with various routes to follow. Pluralism, although a road less travelled, is nonetheless a road worth taking and a road to transformation and innovation in practice.
